# Supplementary material for: The causal relationship between the human gut microbiota and pyogenic arthritis: a Mendelian randomization study
Source: Front Cell Infect Microbiol. 2024 Nov 26;14:1452480. doi: 10.3389/fcimb.2024.1452480 (PMC11629706; doi:10.3389/fcimb.2024.1452480)
Supplement: Supplementary file 1 [file DataSheet1.zip › mendelian test/ebi-a-GCST90027713.csv_leave_one_out.pdf]

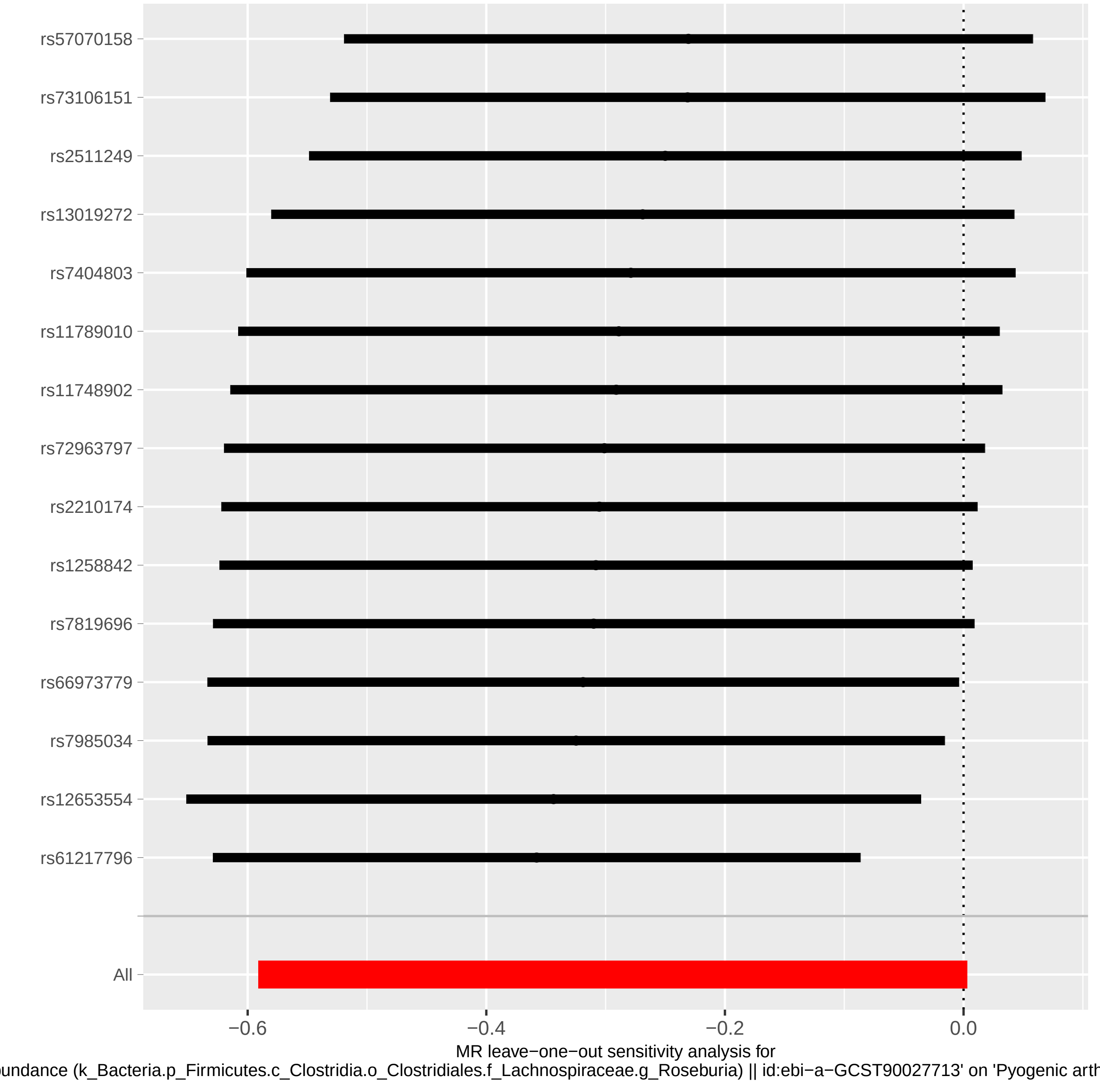

rs57070158
rs73106151
rs2511249
rs13019272
rs7404803
rs11789010
rs11748902
rs72963797
rs2210174
rs1258842
rs7819696
rs66973779
rs7985034
rs12653554
rs61217796
All
−0.6
−0.4
−0.2
0.0
MR leave−one−out sensitivity analysis for
oundance (k_Bacteria.p_Firmicutes.c_Clostridia.o_Clostridiales.f_Lachnospiraceae.g_Roseburia) || id:ebi−a−GCST90027713' on 'Pyogenic arth
